# Nano-Pulsed Laser Therapy Is Neuroprotective in a Rat Model of Blast-Induced Neurotrauma

**DOI:** 10.1089/neu.2017.5249

**Published:** 2018-07-01

**Authors:** Rinat O. Esenaliev, Irene Y. Petrov, Yuriy Petrov, Jutatip Guptarak, Debbie R. Boone, Emanuele Mocciaro, Harris Weisz, Margaret A. Parsley, Stacy L. Sell, Helen Hellmich, Jonathan M. Ford, Connor Pogue, Douglas DeWitt, Donald S. Prough, Maria-Adelaide Micci

**Affiliations:** ^1^ Department of Anesthesiology, University of Texas Medical Branch, Galveston, Texas.; ^2^ Department of Neuroscience and Cell Biology, University of Texas Medical Branch, Galveston, Texas.; ^3^ Center for Biomedical Engineering, University of Texas Medical Branch, Galveston, Texas.

**Keywords:** blast injury, near-infrared light, neuroprotection, non-invasive transcranial laser therapy, optoacoustics, traumatic brain injury

## Abstract

We have developed a novel, non-invasive nano-pulsed laser therapy (NPLT) system that combines the benefits of near-infrared laser light (808 nm) and ultrasound (optoacoustic) waves, which are generated with each short laser pulse within the tissue. We tested NPLT in a rat model of blast-induced neurotrauma (BINT) to determine whether transcranial application of NPLT provides neuroprotective effects. The laser pulses were applied on the intact rat head 1 h after injury using a specially developed fiber-optic system. Vestibulomotor function was assessed on post-injury days (PIDs) 1–3 on the beam balance and beam walking tasks. Cognitive function was assessed on PIDs 6–10 using a working memory Morris water maze (MWM) test. BDNF and caspase-3 messenger RNA (mRNA) expression was measured by quantitative real-time PCR (qRT-PCR) in laser-captured cortical neurons. Microglia activation and neuronal injury were assessed in brain sections by immunofluorescence using specific antibodies against CD68 and active caspase-3, respectively. In the vestibulomotor and cognitive (MWM) tests, NPLT-treated animals performed significantly better than the untreated blast group and similarly to sham animals. NPLT upregulated mRNA encoding BDNF and downregulated the pro-apoptotic protein caspase-3 in cortical neurons. Immunofluorescence demonstrated that NPLT inhibited microglia activation and reduced the number of cortical neurons expressing activated caspase-3. NPLT also increased expression of BDNF in the hippocampus and the number of proliferating progenitor cells in the dentate gyrus. Our data demonstrate a neuroprotective effect of NPLT and prompt further studies aimed to develop NPLT as a therapeutic intervention after traumatic brain injury (TBI).

## Introduction

Over the past two decades, blast-induced neurotrauma (BINT) has become a prevalent health concern due to the increasing incidence of blast-induced traumatic brain injury (TBI) sustained by soldiers in combat.^1–4^ Many victims of closed-head, blast injury experience persistent post-concussive symptoms^5^ and chronic cognitive and emotional deficits secondary to the initial TBI.^6,7^ Although the understanding of TBI pathophysiology has improved, current treatment options for BINT remain limited.

Recently, transcranial low-level laser therapy (LLLT) has gained recognition as an alternative to existing TBI treatments.^8–10^ LLLT uses near-infrared light (600–1000 nm) to stimulate, repair, regenerate, and protect injured tissue. Initial studies of LLLT focused on stimulation of wound healing and reduction of pain and inflammation in various orthopedic conditions.^11^ Recently, several reports demonstrated beneficial effects of LLLT in reducing neuroinflammation, brain lesion volume, and edema in animal models of TBI.^12^ Specifically, they showed a significant neuroprotective effect of transcranial LLLT generated by LED or laser sources (both continuous and pulsed) using controlled cortical impact and closed-head-injury rodent models of TBI. More recently, some clinical case reports tested the therapeutic effect of LLLT for the treatment of chronic TBI patients and showed small but significant improvements in cognitive and motor functions^13–15^; however, as of today, no studies have tested LLLT on animal models of BINT.

In the past few years, new evidence emerged pointing to a potential therapeutic use of non-invasive low-intensity, low-frequency (0.44–0.67 MHz) ultrasound waves for treatment of TBI. Specifically, recent studies have shown that transcranial delivery of low-intensity pulsed ultrasound stimulation reduces brain injury caused by focused ultrasound-induced blood–brain barrier permeablity and reduces edema in a closed-head weight-drop model of TBI.^16,17^

We propose to use optoacoustics to combine the therapeutic effects of light and ultrasound. Optoacoustic waves can be generated in tissues by short (typically, hundreds of nanoseconds or shorter) optical pulses.^18–21^ Absorption of light energy in tissue or any other absorbing medium is followed by temperature rise. Thermal expansion of the irradiated medium induces mechanical stress (pressure rise) upon the condition of stress confinement. This mechanism is referred to as the thermo-optical mechanism of pressure generation. The condition of stress confinement means that there is insignificant stress relaxation in the irradiated volume during the optical pulse. To provide this condition in tissues, the duration of the optical pulse should be shorter than the time of stress propagation out of the irradiated tissue volume.^18–20^ Nanosecond laser pulses can be used to generate conditions of stress confinement for many optoacoustic applications in tissues including the proposed nano-pulsed laser therapy (NPLT).

Such light plus ultrasound combination may be more efficient for therapy than light or ultrasound alone. Moreover, a synergistic effect may be produced when light pulses and light-induced ultrasound (optoacoustic) waves are applied simultaneously resulting in a better therapeutic response.

We developed a novel, medical-grade optoacoustic system for the transcranial delivery of near-infrared light pulses (808 nm) and detection of low-level optoacoustic (ultrasound) waves. This system generates pulses with a duration of 10 nsec, energy of up to 15 mJ, and pulse repetition rate of 20 Hz. It can produce wide-band low-energy optoacoustic waves in tissue via thermoelastic mechanism under stess-confined irradiation conditions. Thus, our system, originally designed to monitor blood oxygenation in the brain^22–24^ has significant therapeutic potential for the treatment of brain injuries because it combines beneficial effects of both near-infrared light and low-level optoacoustic waves.

Here, we test the neuroprotective potential of NPLT when applied transcranially to rats subjected to BINT. We report that transcranial application of NPLT protected from vestibulomotor and cognitive dysfunction, significantly increased the expression of pro-survival genes, reduced the expression of pro-apoptotic genes, and reduced neuroinflammation in the cerebral cortex. Further, NPLT increased proliferation of neural progenitors in the hippocampus, an important brain area for learning and memory.

## Methods

### Animals

Male Sprague-Dawley rats (200–350 g) were used in all experiments described. Experimental protocols were approved by the Institutional Animal Care and Use Committee (IACUC) at the University of Texas Medical Branch, Galveston, in accordance with the guidelines provided by the National Institutes of Health.

### Experimental design and number of animals

**FIG. 1.**
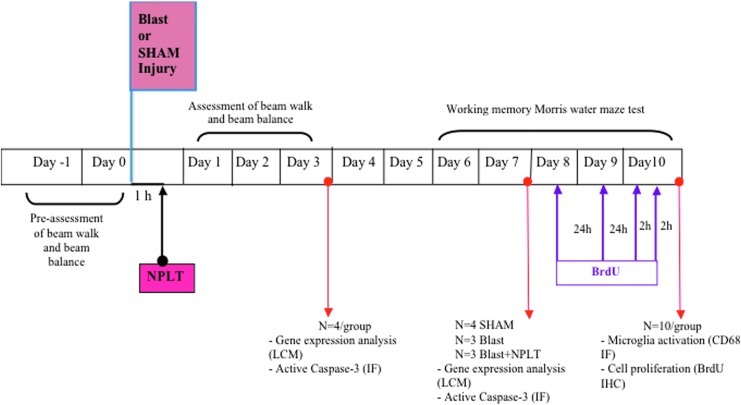
Experimental design. Experimental rats were randomized to receive blast or sham injury. Blast-injured rats were further randomized to receive nano-pulsed laser therapy (NPLT) or no therapy. Vestibulomotor function (beam balance and beam walk) was assessed on post-injury days (PIDs) 1–3 and working memory function (Morris water maze [MWM]) was assessed on PIDs 6–10. On PIDs 3 (*n* = 4/group) and 7 (*n* = 3–4/group), brains were collected for laser-captured microdissection (LCM) of neurons from the cerebral cortex and hippocampus CA1-3 region and for immunofluoerescence analysis (IF) of active caspase-3 and NeuN expression. The expression of select genes in LCM neurons was detected by quantitative real-time PCR (qRT-PCR). On PID 8, 9, and 10 the rats were injected with bromodeoxyuridine (BrdU) for the analysis of cell proliferation. On PID 10, after completion of the MWM test, brains were collected for analysis of microglia activation (CD68 immunofluorescence) and cell proliferation in the hippocampus dentate gyrus (BrdU immunoreacticvity). IHC, immunohistochemistry.

The experimental time line is shown in Figure 1. Rats (*n* = 50) were randomized to receive blast or sham injury. Blast-injured rats were further randomized to receive NPLT or no therapy. All rats were tested for vestibulomotor function on post-injury days (PIDs) 1–3 (18 Sham, 15 Blast, 16 Blast+NPLT). A subset of rats (*n* = 22) were sacrificed on PID 3 (*n* = 4/group) and PID 7 (4 Sham, 3 Blast, 3 Blast+NPLT) for analysis of expression of genes involved in the regulation of cell survival after brain injury.^25^ Neurons were laser-captured from the somatosensory cortex and the CA1-3 region of the hippocampus and quantitative real-time PCR (qRT-PCR) was used to detect messenger RNA (mRNA). Working memory was tested in the remaining rats (*n* = 28) on PIDs 6–10 (10 Sham, 9 Blast, 9 Blast+NPLT). These rats were euthanized on PID 10 for analysis of microglia activation (CD68 immunofluorescence; *n* = 3) and neural stem cell (NSC) proliferation in the hippocampus (bromodeoxyuridine [BrdU] immunoreactivity; *n* = 5). The rationale for looking at different days in the analysis is as follows:

- Vestibulomotor function was analyzed on PIDs 1–3 because it is known to be affected early after injury and to spontaneously recover.
- Working memory was tested on PIDs 6–10 to allow the rats to fully recover their motor function and be able to swim.
- The rationale for studying brain injury-induced gene expression changes on PIDs 3 and 7 was to determine the potential therapeutic efficacy of NPLT to target early molecular events that occur in the first few days following BINT.^25^

### Blast injury

Rats were anesthetized with isoflurane and randomized to receive blast or sham injury. The custom-made Vandenberg device was used to produce blast injury, using nail-gun cartridges inserted into a detachable barrel.^22^ To prevent accidental activation, the device only fires when an operator simultaneously presses two switches, which requires both hands. A solenoid drives a metal bar to strike the firing pin against the cartridge. In this study we used Ramset/Remington nail-gun cartridges of 0.27 caliber with power level 4, which in our unpublished study induced mild-to-moderate brain injury in the animal model. Under these conditions, the Vandenberg blast device produces a combined blast over/underpressure that is followed by a blunt impact caused by the venting gas jet.

**FIG. 2.**
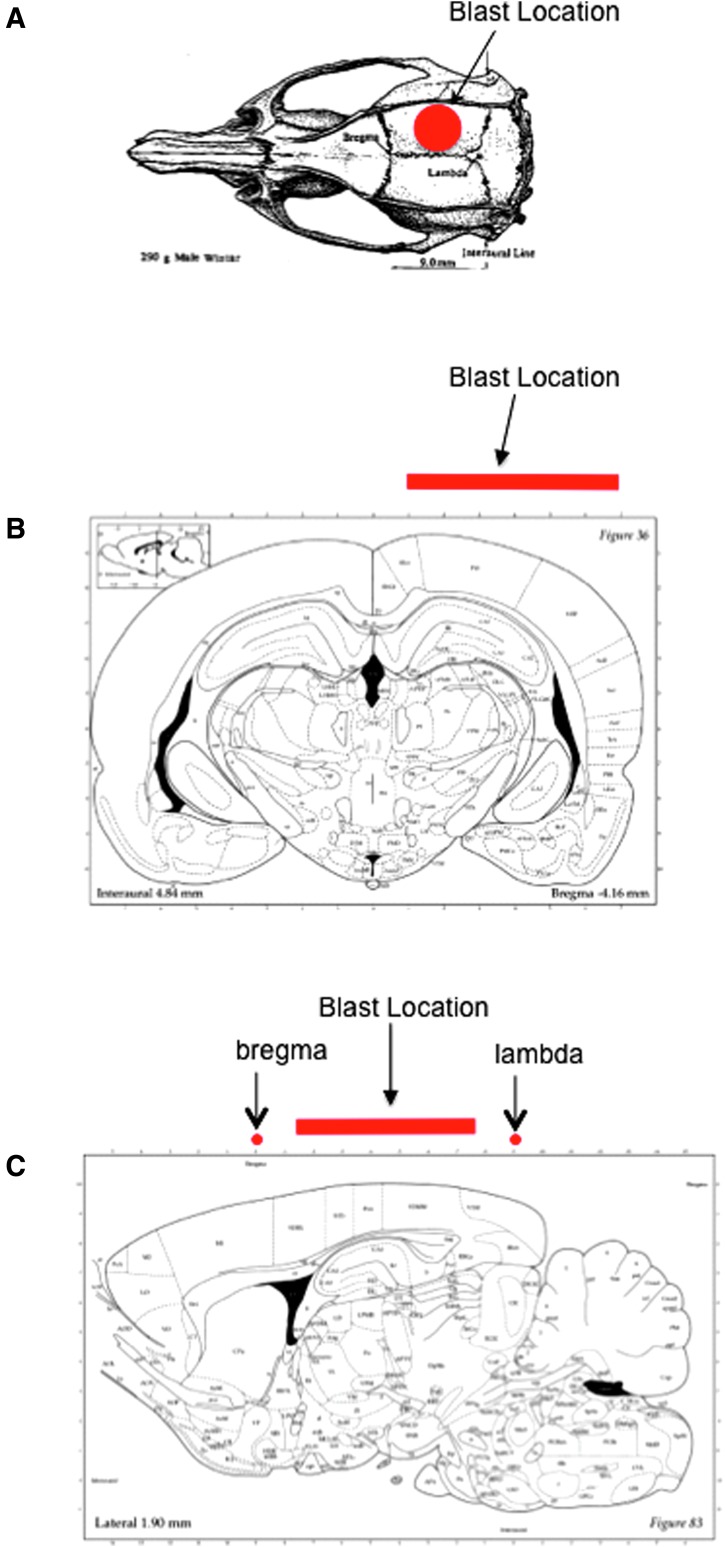
Location of blast injury. **(A)** Dorsal view of the rat skull showing the approximate location of blast injury. **(B)** Coronal view of the rat brain (modified from Paxinos, G., and Watson, C. *The Rat Brain in Stereotaxic Coordinates*, 4th ed. Academic Press: San Diego, Fig. 36) showing the location of the blast at −4.18 mm of bregma (corresponding to the center of the blast location in the coronal plane). **(C)** Sagittal view of the rat brain at 1.90 mm lateral, corresponding to the center of the blast in the sagittal plane. (Modified from Paxinos, G., and Watson, C. *The Rat Brain in Stereotaxic Coordinates*, 4th ed. Academic Press: San Diego, Fig. 83.)

The dorsal surface of the head was shaved and the rat was moved onto a 5-cm thick foam pad to minimize tertiary blast injuries. Using high-speed video recordings, we had previously confirmed that the force of the blast presses the rat into the foam pad. To block both debris (e.g., unburned powder) and heat from reaching the animal, a 1.5-mm thick silicone rubber pad was placed on the head. Earplugs were inserted to protect the eardrums. The rat was positioned under the Vandenberg device, with the opening of the barrel 15 mm above the protective pad and directly over the right hemisphere of the brain. Isoflurane was discontinued and paw pinches were tested repeatedly (once per second) until a withdrawal response was detected, at which point the blank cartidge was fired. For sham injury, the rats were positioned under the blast device but the blank cartridge was not fired. Immediately after the firing of the blank cartridge, rats were removed from the blast device, placed in supine position, and monitored until they recovered the righting reflex. The time to recover the righting reflex was recorded. Rats were then replaced on isoflurane anesthesia and randomized to received NPLT or no therapy. A schematic from the rat atlas indicating the location of the blast injury is shown in Figure 2.

### Nano-pulsed laser therapy

Rats were maintained under isoflurane anesthesia for 1 h until time for the NPLT treatment. NPLT was delivered to the intact rat head through a 3-mm diameter specially developed fiber-optic bundle system positioned as close as possible to the blast location on the head and held in place using a stereotaxic holder. To accurately and consistently treat the area affected by the blast, a circle was drawn on the rat shaved head using a marker to indicate the area directly underneath the Vandenberg device piston. The proprietary, medical-grade, fiber-optic-coupled laser system consists of an optical parametric oscillator that generates short (10 nsec) pulses of near-infrared light (808 nm) at energy of up to 15 mJ and pulse repetition rate of 20 Hz. The pulse energy and spot size on the rat head were 5 mJ and 3 mm, respectively. These pulses generate low-level optoacoustic waves that travel deep into the brain. Rats were treated 1 h after blast injury for a duration of 5 min to provide a dose of 300 J/cm^2^, which is similar to that used for the LLLT with continuous wave light.

### BrdU injections

Experimental rats were injected intraperitoneally with bromodeoxyuridine (BrdU; Sigma Aldrich) at 75 mg/kg daily on PIDs 8–10. On day 10, rats received two injections of BrdU, 2 h apart. Rats were euthanized using isoflurane (4% in anesthetic chamber) followed by decapitation 2 h after the last BrdU injection, and the brains were removed and frozen on dry ice.

### Cognitive and motor function testing

To test the effect of NPLT on blast-induced vestibulomotor and cognitive deficits, rats were subjected to beam walk and beam balance tests on PIDs 1–3 and on the working memory paradigm of the Morris water maze (MWM) on PIDs 6–10 as previously described by Sell and colleagues.^26^

### Vestibulomotor function

#### Beam balance

Rats underwent one training session 24 h before and one pre-assessment test on the day of the blast injury or sham procedure. The rats were trained to balance for 60 sec on a short wooden beam (50 × 1.5 × 4 cm) raised 90 cm off the floor. Once the rats were able to remain on the beam, they were evaluated for three consecutive trials per session and rated using a 6-point scale:

1. Balances with steady posture (grooms, climbs barrier).
2. Balances with unsteady posture (grasps sides of beam and/or has shaky movements).
3. Hugs the beam or slips or spins on the beam.
4. Attempts to balance, but falls off after 10 sec.
5. Drapes over or hangs from the beam, falls off in less than 10 sec.
6. Falls off, making no attempt to balance or hang onto the beam.

#### Beam walk

Animals were trained to traverse a wooden beam (100 × 2.5 × 4.0 cm) elevated 1 m above the floor. Four steel pegs were spaced at equal distances along the top and a darkened goal box was positioned at the far end of the beam. Once trained, the rats were timed during three consecutive trials, with time to reach the goal box as the primary end-point. On the day of injury rats underwent a pre-injury assessment.

### Cognitive function

#### Working memory test

We used the MWM to assess working memory, as described in detail by Sell and colleagues.^26^ Briefly, the experimental animals were placed in a tank filled with water to a level that was 2 cm higher than the hidden platform. Rats were assigned four starting points and four platform locations in a balanced order to avoid starting points too close to the platform. For Trial 1, rats were placed in the tank and allowed 120 sec to find the platform. Once on the platform, the rats were allowed 15 sec to rest and then were placed in the tank again from the same starting point to begin Trial 2. They were again allowed 120 sec to find the platform. Rats were rested 4 min in a heated enclosure before starting a second pair of trials, which used different platform and starting locations. Rats received four pairs of trials daily for 5 consecutive days. All rats received the same sequence of starting points and platform locations.

### Laser capture microdissection (LCM)

On PIDs 3 and 7, rats (4 Sham, 3 BINT, and 3 BINT+NPLT per time-point) were euthanized using isoflurane (4% in anesthetic chamber) followed by decapitation, brain dissection, rapid freezing on dry ice, and storage at −80°C. To prepare for cryo-sectioning, brains were frozen in optimal cutting temperature (OCT) mounting medium, 10-μm coronal brain sections were cut through the hippocampus, between bregma level −3.16 mm and −5.16 mm, on a cryostat (Leica Microsystems CM1850), and mounted on superfrost clean glass slides (Superfrost Plus, Thermo Fisher Scientific Inc., Marietta, OH). To ensure equal representation of sections relative to bregma level, every third section (for a total of 50 sections per brain) was stained with 0.001% Fluoro-Jade C (FJC; Histo-Chem, Inc., Jefferson, AR), a marker for neuronal injury, and counterstained with 1% cresyl violet (a Nissl stain), as previosuly described.^27^ All solutions were prepared with RNase-free water, and the cresyl violet and the FJC were sterile filtered immediately before use.

LCM was performed using a PixCell IIe laser capture microscope with an infrared diode laser (Arcturus Engineering, presently Arcturus/ LifeTechnologies/Thermo-Scientific, Carlsbad, CA). The smallest laser spot size (7.5 μm) was used with a power setting of 75–100 mW and a pulse duration of 0.35–0.75 msec (settings were adjusted, as necessary, for optimum capture of the cells). Uninjured (FJC-negative) neurons from the somatosensory cortex and hippocampus CA1-3 region were captured on a thermoplastic film of separate CapSure Macro LCM caps (Thermo-Fisher Scientific, Carlsbad, CA). Equal sampling was ensured by harvesting FJC-negative cells that were adjacent or in close proximity to FJC-positive cells. In the hippocampus we sampled throughout the CA1-3 region. In the cortex, we sampled throughout the somatosensory cortex. The same number of cells was collected from the selected regions for each experimental animal (600 cells/region/brain). They were placed into a 0.5-mL tube containing 100 μL of Lysis buffer (Ambion/Thermo-Scientific), vortexed, and stored at −80°C until isolation of total RNA.

### RNA isolation, reverse transcription, and quantitative real-time PCR

Total RNA was isolated using the RNA Aqueous Micro kit (Ambion/Thermo Scientific) according to the manufacturer's protocol before DNase treatment at 37°C for 20 min to remove any traces of genomic DNA. The concentration and quality of total RNA was assessed using an Agilent Bioanalyzer with the RNA6000 Pico Lab Chip (Agilent Technologies). Afterward, 1 ng of total RNA was reverse transcribed using the High Capacity cDNA Reverse Transcription Kit (Applied Biosystems/Thermo-Scientific) according to the manufacturer's protocol. Taqman PreAmp Master Mix Kit (Applied Biosystems/Thermo-Scientific) was used to pre-amplify cDNA to increase the quantity of specific cDNA targets: *Bdnf, Stat3, Casp3, Bcl2, Bax, Gapdh*. The cDNA was pre-amplified for 14 cycles with a thermal profile of 1 cycle at 95°C for 10 min and 14 cycles at 95°C for 15 sec/60°C for 4 min. Pre-amplification products were diluted 1:20 with 1X TE buffer before proceeding to quantitative PCR. qRT-PCR was performed on an MX3000P instrument (Agilent Technologies) with Taqman chemistry probes (Applied Biosystems/Thermo-Scientific) with the following thermal profile: 50°C for 2 min (1 cycle), 95°C for 10 min (1 cycle), 95°C for 15 sec/60°C for 1 min (45 cycles). Each PCR reaction was performed in triplicate. Normalization to GAPDH, a housekeeping gene, was performed by calculating the ΔCt for each gene of interest (GOI). This calculation involves subtracting the Ct value of the GOI from the Ct value of GAPDH. All data from the PCR were collected and analyzed by the MXPro software (provided by Stratagene with purchase of Mx3000 Multiplex Quantitative PCR System) and ΔΔCt fold changes were calculated, graphed, and plotted in Excel.

### Tissue processing and immunofluorescence analysis

At 3, 7, or 10 days after BINT or sham injury, rats were euthanized using isoflurane (4% in anesthetic chamber) followed by decapitation, and brains were dissected, rapidly frozen on dry ice, and stored at −80°C. To prepare for cryo-sectioning, tissues were frozen in OCT mounting medium and coronal brain sections (collected in groups of 10 serial sections) were cut through the entire hippocampus, between bregma level −3.16 mm and −5.16 mm, on a cryostat (Leica Microsystems CM1850) and mounted on superfrost clean glass slides (Superfrost Plus, Thermo Fisher Scientific). To ensure equal representation of sections relative to bregma level, every 10th section, for a total of 10 sections per brain, was selected for immunofluorescence analysis.

#### Analysis of microglia activation

Coronal brain sections (14 μm) were fixed in ice-cold 10% buffered formalin and incubated in phosphate buffered saline (PBS) containing 10% normal goat serum and 0.3% Triton X-100 for 30 min at room temperature. The sections were incubated with primary antibodies diluted in PBS containing 1.5% normal goat serum overnight at 4°C. They were then incubated with secondary antibodies (594-Alexa-conjugated, Invitrogen Co., Carlsbad, CA; 1:400 dilution in PBS with 1.5% normal goat serum) for 1 h at room temperature. After washing in PBS, the sections were rinsed in tap water and coverslipped with mounting media containing DAPI (Vector Laboratories, Burlingame, CA).

#### Analysis of active caspase-3

The same rat brains used for LCM on PIDs 3 and 7 were used for immunofluorescence analysis of active caspase-3. Coronal brain sections (10 μm) were fixed in ice-cold 10% buffered formalin and incubated in PBS containing 10% normal goat serum and 0.3% Triton X-100 for 30 min at room temperature. The sections were incubated with primary antibodies (rabbit anti-cleaved caspase-3, 1:200; R&D; and mouse anti-NeuN, 1:100; Millipore) diluted in PBS containing 1.5% normal goat serum overnight at 4°C. They were then incubated with secondary antibodies (594- and 488-Alexa-conjugated, Invitrogen Co., Carlsbad, CA; 1:400 dilution in PBS with 1.5% normal goat serum) for 1 h at room temperature. After washing in PBS, the sections were rinsed in tap water and coverslipped with mounting media containing DAPI (Vector Laboratories, Burlingame, CA).

#### Analysis of cell proliferation

For BrdU detection, after fixation in ice-cold 10% formalin for 30 min, the sections were washed in 0.1 M PBS (pH 7.4), incubated in 1N HCl solution for 30 min at 37°C and then washed in 0.1 M borate buffer (pH 8.5). Endogenous peroxidase activity was blocked by incubation in 1.5% hydrogen peroxide in dH_2_O for 30 min. The sections were blocked and permeabilized in 5% normal goat serum and 0.3% triton X-100 in PBS for 30 min. This step was followed by incubation with the primary antibody (ms anti-BrdU; 1:100 DAKO) in PBS overnight at 4°C. The following day, after washing in PBS, sections were incubated with secondary antibody (biotinylated goat anti-mouse, ABC Mouse Kit, Vector Laboratories) before incubation with an avidin-biotin complex (ABC Kit, Vector Laboratories) according to the manufacturer's instructions. Staining was visualized by incubating the sections with DAB substrate for 10 min. After washing for 5 min in tap water, a light counterstain using Hematoxylin QS (Vector Laboratories) was performed. Sections were dehydrated in graded ethanol solutions of increasing concentrations, cleared in xylene, and coverslipped with Permount (Sigma). For double immunofluorescence for BrdU and cell-specific markers (NeuN to label neurons and GFAP to label astrocytes) sections were fixed in ice-cold 10% buffered formalin, incubated in PBS containing 10% normal goat serum and 0.3% Triton X-100 for 30 min at room temperature, incubated in 1N HCl solution for 30 min at 37°C, and then washed in 0.1 M borate buffer (pH 8.5). The sections were incubated with primary antibodies (ms anti-BrdU; 1:100 DAKO + rabbit anti-NeuN; 1:100 Millipore, or ms anti-BrdU; 1:100 DAKO + rabbit anti-GFAP; 1:1000 DAKO) diluted in PBS containing 1.5% normal goat serum overnight at 4°C. They were then incubated with secondary antibodies (594- and 488-Alexa-conjugated, Invitrogen Co., Carlsbad, CA; 1:400 dilution in PBS with 1.5% normal goat serum) for 1 h at room temperature. After washing in PBS, the sections were rinsed in tap water and coverslipped with mounting media containing DAPI (Vector Laboratories).

Images were taken with an Olympus BX51 microsope equipped with a cooled CCD camera.

### Quantification of immunohistochemical staining

Two independent investigators, who were blinded to the experimental groups, quantified immunopositive cells in every 10th section of a series of coronal brain sections from −3.15 mm to −5.16 mm of bregma for a total of 10–15 sections per brain. For active caspase-3 quantification, double immunofluorescence was used to co-localize active caspase-3 and NeuN in the same sections. Only cells displaying an intact nucleus positive for NeuN that were also positive for active caspase-3 were counted. Active Casapse3^pos^/NeuN^pos^ cells and CD68^pos^ cells in each section were viewed using ImageJ software and the total number of cells in the region of interest was obtained. The inclusion and exclusion criteria were as follows: For active caspase-3, only cells with a nucleus positive for NeuN were counted; for CD68, only cells with an intact nucleus (identified by DAPI) and positive for CD68 were counted. The mean of the two investigators' counts was calculated to get a total mean count for each brain. Values from each animal (three per group) were averaged to calculate the mean number for the group.

To quantify proliferating cells in the hippocampus dentate gyrus, a total of 15 sections per brain at the level of the dentate gyrus from −3.16 mm to −5.16 mm of bregma were examined by two independent investigators who were blinded to the experimental groups. Specifically, the subgranular zone (SGZ, defined as a two cell-body thick layer between the hilus and granule layer) was outlined and individual BrdU^pos^ cells with stained nuclei were counted in the outlined area. The reported number of BrdU^pos^ cells in the results section represents the average number of cells for all counted sections. The mean of the two investigators' counts was calculated to get a total mean count for each brain. Mean values were then calculated for each group (five per group).

### Statistical analysis

Statistical analysis was performed with the aid of a proprietary software (GraphPad Prism 7). For beam walk and beam balance tests, differences between groups were determined by analysis of variance (ANOVA) and post hoc Fisher's test. For gene expression analysis and quantitative analysis of immunohistochemistry, group differences in the mean values were evaluated by ANOVA and post hoc Tukey's multiple comparisons test. Values are expressed as mean ± standard error of the mean (SEM). Differences were considered significant when *p* < 0.05.

## Results

### Transcranial application of NPLT prevented blast-induced vestibulomotor and cognitive dysfunctions

**FIG. 3.**
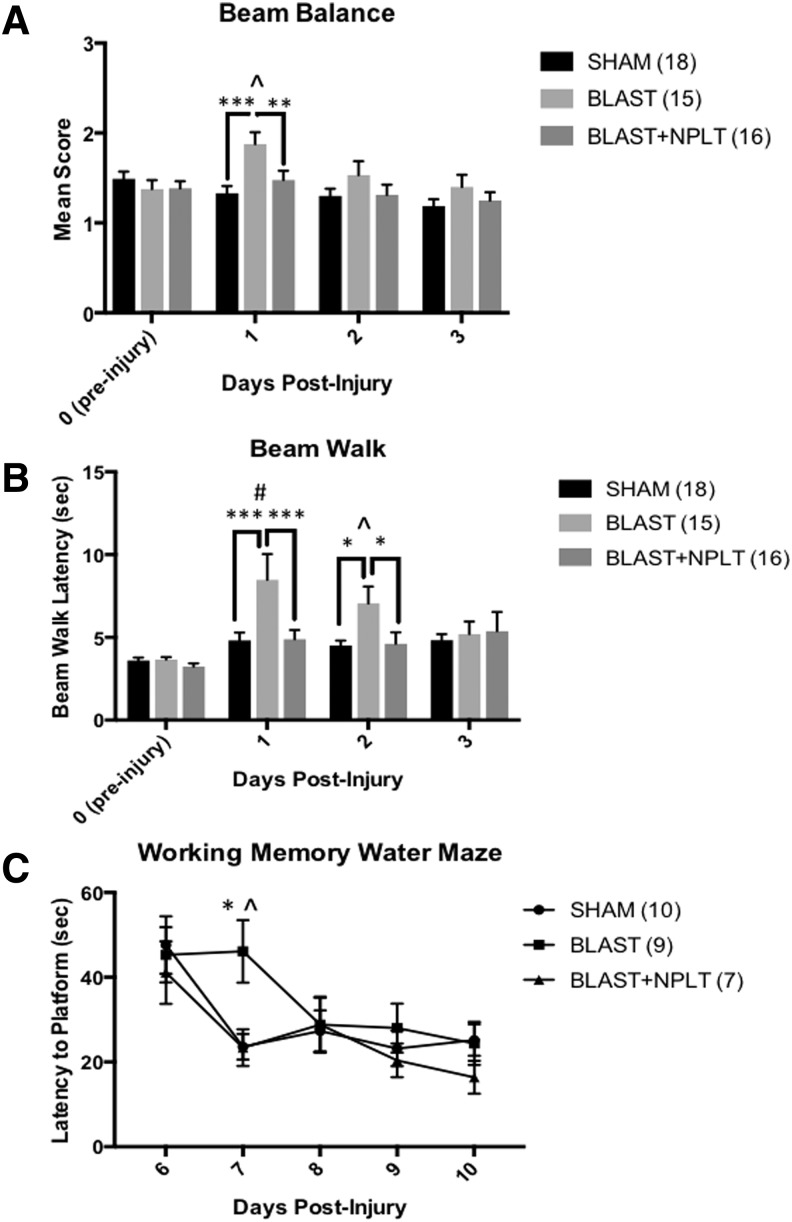
Nano-pulsed laser therapy (NPLT) prevents blast-induced vestibulomotor and cognitive dysfunctions. **(A)** The beam balance test: Rats that received blast injury had higher scores on post-injury day (PID) 1 compared with rats that received sham injury or blast plus NPLT treatment. Two-way analysis of variance (ANOVA) revealed a significant overall effect of treatment (*p* < 0.001) and time after injury (*p* < 0.001); Post-hoc Fisher's multiple comparisons test revealed ***p < 0.001 BLAST vs. SHAM; **p < 0.01 BLAST vs. BLAST+NPLT on PID 1; and ^*p* < 0.01 BLAST PID1 vs. Blast PID 0 (pre-injury). The number of rats in each experimental group is shown in parenthesis. **(B)** The beam walk test: rats that received blast injury showed longer latencies to traverse the beam on PIDs 1 and 2 compared with rats that were sham injured or received blast plus NPLT treatment. Two-way ANOVA revealed a significant overall effect of treatment (*p* < 0.01). Post-hoc Fisher's multiple comparisons test revealed ***p < 0.001 BLAST vs. SHAM on PID 1 and *p < 0.05 BLAST vs. BLAST+NPLT on PID 2; and #*p* < 0.0001 BLAST PID 1 vs. BLAST PID 0 (pre-injury) and ^*p* < 0.01 BLAST PID 2 vs. Blast PID 0 (pre-injury). The number of rats in each experimental group is shown in parenthesis. **(C)** The working memory water maze test: Rats were tested in the working memory paradigm of the Morris water maze test on PIDs 6–10. Only data from Trial 2 are shown. Two-way ANOVA revealed a significant overall effect of treatment (*p* < 0.05) and of time after injury (*p* < 0.0001); Post hoc Tukey's multiple comparisons test revealed **p < 0.01 BLAST vs. SHAM and ^*p* < 0.05 BLAST vs. BLAST+NPLT on PID 7. Data are mean ± standard error of the mean (SEM). Number of animals is shown in parenthesis.

Rats that received BINT scored significantly worse on the beam balance test on PID 1 as compared with sham controls (*p* < 0.001). Further, NPLT-treated blast-injured rats received scores similar to sham rats and significantly better than untreated blast-injured rats (*p* < 0.01 Blast vs. Blast+NPLT; Fig. 3A). On the beam walk test, injured rats took significantly longer to cross the beam on PIDs 1 and 2 as compared with both sham controls (*p* < 0.001) and Blast+NPLT-treated rats (*p* < 0.05; Fig. 3B). On the MWM (working memory paradigm), untreated, injured rats (Blast) took significantly longer on PID 7, Trials 2, to find the platform compared with the Blast+NPLT (*p* < 0.05) and sham rats (*p* < 0.01; Fig. 3C).

### Transcranial application of NPLT upregulated neuroprotective genes in cortical neurons after blast-induced neurotrauma

**FIG. 4.**
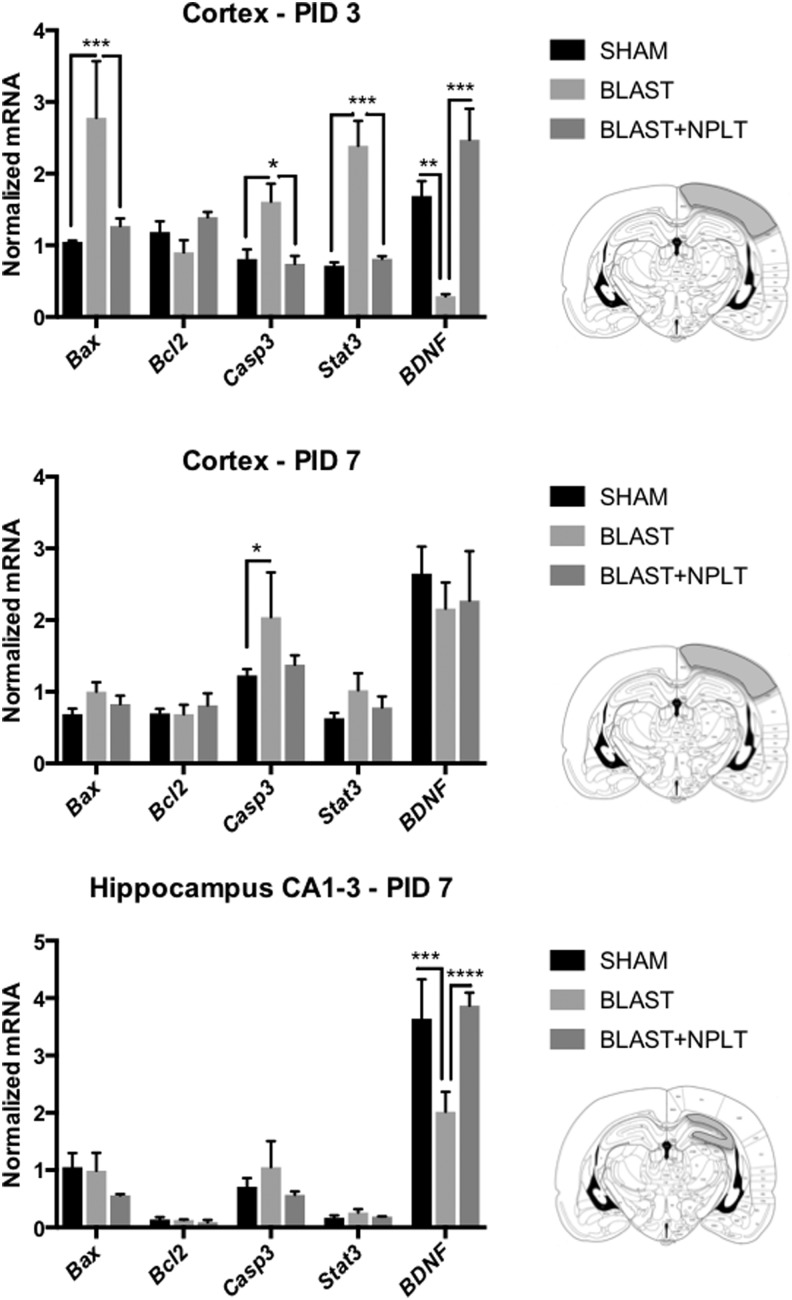
Nano-pulsed laser therapy (NPLT) prevents early blast-induced changes in expression of genes involved in the control of cell death and survival in the cortex and hippocampus. Experimental animals were euthanized on post-injury days (PIDs) 3 **(A)** and 7 **(B,C)**, and the brains removed, stained with Fluoro-Jade to identify injured cells, and counterstained with cresyl violet (CV). Laser-capture micro-dissection (LCM) was used to collect cortical neurons in the somatosensory cortex (A,B) and pyramidal neurons in the CA1-3 region of the hippocampus (C). Only Fluoro-Jade^neg^/CV^pos^cells were captured. The expression of select messenger RNAs (mRNAs) was measured by quantitative real-time PCR (qRT-PCR) analysis. Data were normalized to GAPDH expression and expressed as mean ± standard error of the mean (SEM); *n* = 4 for SHAM, *n* = 3 for BLAST and BLAST+NPLT. *p < 0.05; **p < 0.01; ***p < 0.001; ****p < 0.0001, two-way analysis of variance (ANOVA) followed by Tukey's multiple comparisons test.

To determine whether NPLT can reduce neuronal injury in the early days after the blast insult, we used qRT-PCR and LCM to measure the expression of select mRNA known to be involved in neuronal injury and survival. Specifically, sections were stained with FJC (a marker of neuronal injury) and only FJC^neg^ neurons (identified by cresyl violet staining) adjacent to FJC^pos^ cells were laser captured from the somatosensory cortex between −3.16 mm and −5.16 mm of bregma and from the hippocampus CA1-3 region. We measured mRNA encoding for the following genes: the neurotrophic factor BDNF, the transcription factor STAT3 (known to be upregulated in response to injury), the anti-apoptotic protein BCL2, and the pro-apoptotic proteins BAX and CASPASE-3. We found that in FJC^neg^ cortical neurons isolated on PID 3 from blast-injured rats, the mRNA levels for BAX, CASPASE-3, and STAT3 were significantly greater compared with both sham and Blast+NPLT rats, whereas the levels of BDNF were significantly lower (Fig. 4A). At 7 days after blast-injury the expression of these genes had returned to normal in cortical neurons (Fig. 4B). On the other hand, in the hippocampus CA1-3 region BDNF was significantly reduced 7 days after injury, but had retruned to sham levels in the Blast+NPLT-treated rats (Fig. 4C).

**FIG. 5.**
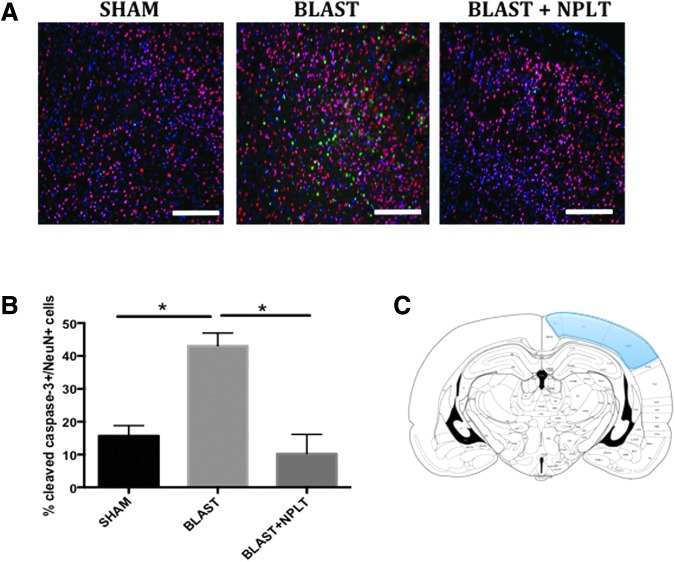
Activation of caspase-3 is reduced after nano-pulsed laser therapy (NPLT) treatment in blast-injured brains. Immunofluorescence analysis from rats euthanized on post-injury day (PID) 3. **(A)** Representative images of brain sections at the level of the somatosensory cortex stained with an antibody against the neuronal marker NeuN (red) and an indicator of apoptotic cell death, active caspase-3 (green). Nuclei are counterstained with DAPI and are shown in blue. Calibration bar is 50 μm. **(B)** Quantitative analysis of the number of active caspase-3^pos^/NeuN^pos^ cells. *N* = 3 rats/group. *p < 0.05 one-way analysis of variance (ANOVA) followed by Tukey's multiple comparisons test. **(C)** Modified from Paxinos, G., and Watson C. *The Rat Brain in Stereotaxic Coordinates*, 4th ed. Academic Press: San Diego, Fig. 35.

To determine whether NPLT resulted in reduced apoptotic cell death after blast injury, we used immunofluorescence to examine the expression of the active form of caspase-3 (indicative of the activation of an apoptotic cascade of events that leads to cell death) in neuoronal cells (identified by the expression of the neuronal marker NeuN). We found that in blast-injured rats, active caspase-3 immunoreactivity was present in the somatosensory cortex between −3.16 mm and −5.16 mm of bregma (an area directly underneath the blast; Fig. 5A). Moreover, we found that NPLT significantly reduced the number of active caspase-3^pos^ neurons 3 days after blast injury as compared with untreated blast-injured rats (*p* < 0.05 Blast vs. Blast+NPLT; Fig. 5B). On the other hand, active caspase-3 was not detected by immunofluorescence in the brain 7 days after blast injury (data not shown).

### Transcranial application of NPLT reduced microglia activation after blast-induced neurotrauma

**FIG. 6.**
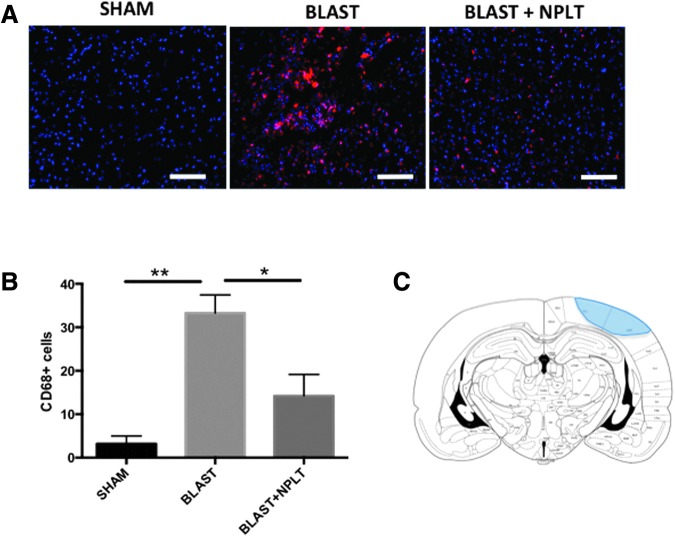
Nano-pulsed laser therapy (NPLT) decreases microglia activation 10 days after blast injury. **(A)** Representative images of brain sections at the level of the somatosensory cortex (bregma level: −4.16 mm) stained with an antibody against CD68 (a marker of activated microglia; red). Nuclei are counterstained with DAPI and are shown in blue. Calibration bar is 50 **μ**m. **(B)** Quantification of the number of CD68^pos^ cells in the cortex. *N* = 3 rats/group. *p < 0.05; **p < 0.01 one-way analysis of variance (ANOVA) followed by post hoc Tukey's multiple comparisons test. **(C)** Representative coronal view of the rat brain at −3.80 mm of bregma showing the location of CD68^pos^ cells (shaded area) identified by immunofluorescence in the brain of BLAST and BLAST+NPLT rats (modified from Paxinos, G., and Watson, C. *The Rat Brain in Stereotaxic Coordinates*, 4th ed. Academic Press: San Diego, Fig. 35).

The onset of neuroinflammation is a major hallmark of the acute and chronic sequelae of brain injury. We analyzed inflammation 10 days after blast injury by immunofluorescence using a specific antibody against CD68, a marker of activated microglia. Numerous CD68^pos^ cells were observed in the somatosensory cortex of blast-injured rats between −3.16 mm and −5.16 mm of bregma (an area directly underneath the blast), whereas few or no CD68^pos^ cells were observed in both sham and blast-injured rats that received NPLT (Fig. 6A). Quantification studies confirmed that the number of CD68^pos^ microglial cells was significantly lower in the somatosensory cortex of NPLT-treated blast-injured rats as compared with untreated blast-injured rats (*p* < 0.05 Blast vs. Blast+NPLT; *p* < 0.01 Blast vs. Sham; Fig. 6B).

### Transcranial application of NPLT stimulated proliferation of neuronal progenitors in the hippocampus

**FIG. 7.**
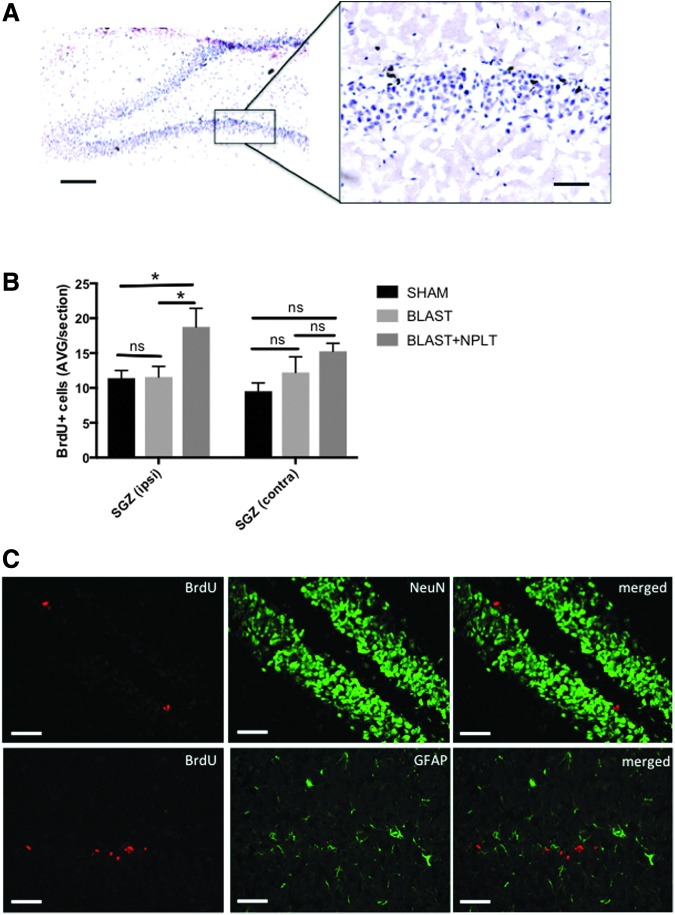
Nano-pulsed laser therapy (NPLT) increases cell proliferation in the SGZ of the hippocampus dentate gyrus 10 days after blast injury. **(A)** Representative images of bromodeoxyuridine (BrdU) incorporation in the subgranular zone (SGZ) of the dentate gyrus of the hippocampus. BrdU is shown in brown. Hematoxylin was used to counterstain nuclei (shown in blue). Calibration bars are 100 μm and 50 μm (insert). **(B)** Quantitative analysis of the number of BrdU^pos^ cells in the SGZ on post-injury day (PID) 10. *p < 0.05; two-way analysis of variance (ANOVA) followed by Tukey's multiple comparisons test. *N* = 5 rats/group. **(C)** Representative images of brain sections at the level for the SGZ of hippocampus dentate gyrus stained with antibodies against BrdU, NeuN (a neuronal marker), and GFAP (a marker for astrocytes). Calibration bar is 50 μm.

Throughout life, proliferating neuronal progenitor cells (NPCs) in the subgranular zone (SGZ) of the hippocampus dentate gyrus generate new granule cells that incorporate in the hippocampal circuitry.^28–30^ We measured cell proliferation in the SGZ using BrdU incorporation (Fig. 7A). BrdU incorporation is commonly used to assess the number of NPCs in the hippocampus dentate gyrus because multiple studies, using BrdU immunohistochemistry and confocal microscopy, have revealed that BrdU-incorporating cells in the SGZ express markers of NPCs.^31–37^ Our quantitative analysis of BrdU immunohistochemistry revealed that, 10 days after blast injury, NPLT significantly increased the number of BrdU^pos^ cells in the ipsilateral SGZ, as compared with blast-injured (*p* < 0.05, Blast+NPLT vs. Blast) and sham rats (*p* < 0.05, Blast+NPLT vs. Sham), but not in the contralateral SGZ (Fig. 7B). We further confirmed that BrdU^pos^ cells in the SGZ were negative for the neuronal marker NeuN and for GFAP (a marker for astrocyes; Fig. 7C), further supporting the fact that these cells are undifferentiated progenitor cells.

## Discussion

In this study, we used the Vandenberg device to produce BINT in adult rats. This established rodent model of blast injury is known to reproduce many features associated with the clinical sequelae experienced by both military personnel and civilians exposed to high pressure waves generated by explosive devices. Although the manner through which blast energy is transmitted to the brain remains controversial, one of several theorized mechanisms of primary blast injury is the transfer of kinetic blast energy to the cerebral vasculature and brain via the great vessels of the thorax.^38–43^ The results of studies in which protecting the chest from blast exposure reduced brain injury support this mechanism.^44,45^ In contrast, in a study in which pigs were outfitted with a lead-and-foam-lined vest that covered the chest and upper abdomen, significant increases in intraparenchymal and intravascular pressures were observed.^46^ Apnea, meningeal bleeding (ferrets), and multi-focal subdural and subarachnoid hemorrhages (rabbits) were observed after blast injury in ferrets^47^ and rabbits^48^ with thoracic and abdominal protection. These results of blast-induced brain injury in the presence of thoracic protection indicate that primary blast exposure to the head in the absence of thoracic injury is sufficient to produce significant brain injury. Here, we used head-only blast exposure to identify primary blast effects to the head alone, excluding the possibility of indirect brain injury through thoracic transmission of the blast wave.

Using this model we tested the therapeutic potential of a novel proprietary system for non-invasive transcranial delivery of short laser pulses of near-infrared light that generate optoacoustic waves.

Photobiomodulation and LLLT utilize either continuous wave (CW) light or long (tens of milliseconds) pulses that do not provide the stress-confined condition and cannot generate optoacoustic waves. Many groups have been studying photobiomodulation and LLLT at different CW light parameters.^8–10,13–15,49,50^ Recent reviews (and references therein) discuss the progress achieved with CW or long pulses in photobiomodulation and LLLT.^12,51^

Light pulses with duration shorter that 10 nsec (for instance, picosecond or femtosecond pulses) can be used for the NPLT as well because they too generate optoacoustic waves in tissues.^18–20^ Moreover, pulses with durations of the order of hundreds of nanoseconds produce optoacoustic waves with high efficacy in tissues.^21^ Therefore, an NPLT therapeutic effect can be achieved by pulses with durations in a range from femtoseconds to microseconds.^18–20^

Another important aspect of NPLT is that, although pulse energy and average power used in our study are low, the short pulses generated have high peak intensity. This may produce multi-photon effects including two-photon photochemical and photobiological effects.^52^ Moreover, irradiation by short pulses of tissue micro-volumes with higher absorption may result in local heating of these volumes.^53^ Moderate local heating by the laser pulses used in our study may have biomodulation/therapeutic effects as well. All these and the optoacoustic effects of short optical pulses on biomodulation/therapy should be further studied.

Other devices deliver LLLT that utilize either continuous wave light or long (tens of milliseconds) pulses that do not provide the stress-confined conditions necessary to generate optoacoustic waves in tissue.^18^ In this study, we used short laser pulses to generate optoacoustic waves; thus, the injured tissue was irradiated by near-infrared light and ultrasound (optoacoustic) waves. Because low-level ultrasound has therapeutic effects,^16,17,54^ both light and ultrasound can contribute to the neuroprotective effects produced by NPLT. Moreover, the light pulses and optoacoustic waves may have synergistic effects that can result in more efficient neuroprotection. Also, optoacoustic waves travel deeper into the brain than near-infrared light, which has been shown to reach a depth of only 40–50 mm in the human brain.^55^ The deeper penetration by NPLT may result in a stronger therapeutic effect as compared with LLLT. Our data show that transcranial application of NPLT delivered 1 h after blast exposure improved vestibulomotor and cognitive function. Previous reports have shown that transcranial delivery of low-level near-infrared light generated by LED or laser sources improved the performance of experimental animals subjected to memory-related tasks in rodent models of TBI.^51,56^ Our report is the first to test the effect of a therapy that combines low-level near-infrared light and ultrasound (optoacoustic) waves in a model of BINT. Specifically, our data demonstrate the ability of transcranial NPLT to reduce both vestibulomotor and cognitive dysfunctions that represent a common and severely debilitating clinical manifestation in blast-exposed individuals.

Perhaps these functional benefits are due to protective effects of NPLT on gene expression early after injury. For example, the pro-apoptotic genes (Bax and caspase-3) were upregulated and a neuroprotective gene (Bdnf) was downregulated in cortical neurons 3 days after injury. NPLT prevented these damaging changes in gene expression. Additionally, 3 days after BINT plus NPLT treatment, significantly fewer cortical neurons expressed activated caspase-3. Although caspase-3 activation per se might not always result in cell death, numerous brain injury studies use activation of caspase-3 as an indicator of neuronal damage; and increased expression of pro-survival genes such as BDNF and Bcl-2 as indicators of neuroprotection.^25,27,57–64^ Therefore, our data point to a neuroprotective effect of NPLT, when applied early after BINT.

Further, we show that the protective effect of NPLT is not limited to the most external cortical structures but extends to deeper structures of the brain, such as the hippocampus. The hippocampus plays a critical role in memory and learning, and is one of the brain areas most affected by TBI.^27^ An important neurotrophin known to support hippocampal neurons is Bdnf. Our results indicate that NPLT treatment protected against injury-induced decreases in Bdnf gene expression in the hippocampus 7 days after blast injury. These results, along with the improved performance of NPLT-treated rats in the working memory water maze test, indicate a therapeutic role for NPLT in preserving hippocampal neurons as well as hippocampal-dependent cognitive functions after BINT.

The hippocampus is one of the brain areas in which neurogenesis is known to occur throughout life.^65^ NPCs located in the SGZ of the dentate gyrus of the hippocampus proliferate and migrate within the granular cell layer, where they become mature neurons. Much evidence in the literature points to a critical role for neurogenesis in supporting cognitive function. Specifically, whereas a reduction in the numbers of NPCs in the dentate gyrus is associated with impaired hippocampus-dependent functions (learning and memory),^66–68^ an increase in NPCs contributes to improved learning and memory.^69–72^ The incorporation of BrdU (an analogue of thymidine) into newly synthetized DNA of proliferating cells in the SGZ is an established method to assess neurogenesis in the dentate gyrus.^37^ Here we show that NPLT increases the number of proliferating (BrdU^pos^) cells in the SGZ of rats 10 days after blast injury. Although we did not perform double label staining using specific markers of neuronal progenitor cells, numerous evidence in the literature^31–36^ strongly suggest that BrdU immunoreactivity in the SGZ represents NPCs and prompts further studies aimed at characterizing the effect of NPLT on neurogenesis, particularly whether NPLT-mediated increase in cell proliferation in the SGZ results in increased number of granular neurons in the hippocampus dentate gyrus.

Another important result of our work is the reduction of the inflammatory response after BINT observed in NPLT-treated rats. Activation of microglia is a well-known sequelae of brain injury in humans associated with cogntive dysfunctions and early onset of neurodegenerative disorders.^73^ Our data show that 10 days after blast injury, activated CD68^pos^ microglia cells are significanly less abundant in the cortex of rats treated with NPLT as compared with untreated blast-injured rats. Inflammation mounts in the hours and days following brain injury in response to cell damage and, in our study, NPLT was administered 1 h after blast; therefore, it is possible that the reduction in inflammation that we see after 10 days is a consequence of neuroprotective properties rather than a direct anti-inflammatory effect of NPLT. Future studies will be needed to determine whether NPLT can reduce inflammation when administered in the days to weeks following intital TBI, after microglia have already been activated.

In conclusion, we report, for the first time, that transcranial application of a novel NPLT, combining the benefits of both near-infrared light and low-intensity ultrasound waves, has the following beneficial effects in a rodent model of BINT: It significantly reduces neuronal cell death and neuroinflammation, increases both neurotrophin expression and proliferation of neural progenitors in the hippocampus, and provides significant vestibulomotor and cognitive improvements. Due to increased penetration in the brain of the ultrasound component, NPLT has greater translational value for the treatment of TBI survivors compared with near-infrared light alone and prompts further studies to test this promising therapy. The results presented in this study are just the beginning of our investigation into the neuroprotective effects of NPLT after a blast injury. The effects of NPLT on the chronic effects of BINT are beyond the scope of this study. Nevertheless, it is possible that mitigating the early effects of a brain injury may prevent or reduce the progression of the secondary effects of the injury.
